# Cost objects: How is your ED performing?

**DOI:** 10.1186/s12913-020-05384-2

**Published:** 2020-06-17

**Authors:** Verónica Fuentes-Cáceres, Liliana Neriz, Alicia Núñez-Mondaca, Ricardo Mateo

**Affiliations:** 1 School of affiliation: Department of Management Control and Information Systems, School of Economics and Business, Universidad de Chile, Zip code: 8330015, Diagonal Paraguay 257, office, 2004 Santiago, Chile; 2 School of affiliation: Business Department, University of Navarra, Pamplona, Navarra Spain

**Keywords:** Costs and cost analysis, Emergency service, hospital, Cost allocation, Process assessment

## Abstract

**Background:**

The aim of this study is to a propose a standardized methodology to identify a list of cost objects that can be used by any ED to compute costs considering that the resulting data must facilitate unit management by improving the information available for decision-making.

**Methods:**

This study considers two stages, first, we analyzed the case-mix of two hospitals collecting their data to define and diagram their processes, activities and to obtain their cost objects, second, we used four additional hospitals to validate our initial findings.

**Results:**

We recognized 59 cost objects. Hospitals may have all these cost objects or just a subset of them depending on the services they provide.

**Conclusions:**

Among the main benefits of our cost objects definition are: the possibility of tracing the processes generated by the services delivered by EDs, the economic sense in its grouping, the chance of using any costing methodology, the flexibility with other classification systems such as DRGs and ICDs, and the opportunity of costing for both diseases and treatments. Furthermore, cost comparison among hospitals using our final 59 cost objects list is more accurate and based on comparable units. In different EDs, each cost object will be the result of a similar combination of activities performed. We also present the results of applying this cost objects list to a particular ED. A total of 53 out of 59 cost objects were identified for that particular unit within a calendar year.

## Background

Emergency care expenditures are a growing problem both in Chile and worldwide. The level of emergency care spending in the United States is between 5 and 6% of the total health expenditure, reaching 10% in some states [1]. However, it is still unclear how much it costs to deliver emergency care worldwide. The reason is twofold. Firstly, cost calculation of clinical processes of Emergency Departments (EDs) does not allow comparison between healthcare units or services. Secondly, no standardized categorization for grouping costs has been defined, i.e. cost objects. A cost object is anything for which a separate measurement of costs is desired [2]. In health care, cost objects could be patients, products, projects, service contracts, and any other work unit [3].

### Cost objects

Different published studies use various types of cost objects for EDs. For example, cost objects of an ED can be classified into three types based on the patient’s status: Urgency; Emergency and Non-Emergency [4]. This definition addresses the classification of patients from a more macro perspective. Other key cost objects that have been used for allocation are: Diagnostic Related Groups (DRGs) [5] and case-mix by using the international classification of diseases (ICD) [6]. These two classifications do not provide complete information about ED services, since they were designed to facilitate billing considering ED charges according to the acuity level of the patient and the intensity of supplies and services provided, so most of the time EDs are seen as an intermediate service.

Other studies consider cost objects just for a subset of ED cases, such as: division costs for services to specific patients, for example hospitalized patients [7] and pre-selected diseases [8]. Another methodology to compute the costs of the clinical processes in EDs, uses homogeneous functional groups, which are defined based on similar consumption activities followed by an imputation to clinical processes [9]. However, the authors do not present the cost objects and suggest that each hospital should code their own diseases according to the ICD. Similarly, other authors define cost objects clustering as groups whose services similar demands for ED functions (i.e. ambulatory patient groups, physicians’ current procedural terminology groups) [10].

Other studies use cost centers related to patients [11]. However, the study does not distinguish the different services and processes carried out by the ED. It can be observed that there is still no consensus on how to classify cost objects for EDs. In fact, the choice of cost objects is difficult given the variety of services provided, yet very important. In this study we consider that a correct definition of cost objects should aim to measure the costs of either treatments or diseases if needed, and trace the processes and services provided by the ED considering the whole set of processes and activities the ED perform not just a subset.

Cost objects have enormous financial importance because they are the foundations of health insurance billing, and thus are tied to health systems financing. The lack of standardization and homogeneity in defining cost objects make comparing among units/services difficult and sometimes even impossible. Therefore a common definition of cost objects is required, in other words the same vocabulary for recording, reporting and monitoring emergency health problems. Thus, this paper aims to create a standardized methodology to identify a list of cost objects for EDs that can be used as a starting point to collect feasible data for decision-making purposes and to provide global data for analysis such as tracing processes, computing costs and allowing comparisons among other ED hospitals. Additionally, in order to test the viability of implementing this list of cost objects with a costing methodology, in this particular case we use the activity-based costing (ABC) method.

In the healthcare sector, there have been attempts to apply the ABC costing methodology since the 1990s due to the need to have more accurate and practical costing systems for a more effective cost control **[**12**–**14**]**. There are some experiences in EDs arguing that this methodology allows for a better imputation of costs, eases processes monitoring, allows inter and intra-hospital comparisons, provides more realistic information and adapts better to the clinical decision process based on protocols **[**9**,**^15^**]**. Also, another study computed the total cost of patient care in an ED using Time-Driven Activity-Based Costing (TDABC) **[**16**]**.

The next section presents the methodology used to achieve these purposes.

## Methods

In order to determine cost objects in EDs, this study considers two stages and a sample including a total of six hospitals. The first stage includes both on-site observations and interviews conducted in two different EDs. To ensure a more representative sample, the first hospital selected (hospital 1 in Table 1) is located in Santiago (the Chilean capital), while the second hospital selected is located in a smaller urban city (hospital 2 in Table 1). The observation and interviewing process lasted approximately 3 months in each ED, and as a result, diagrams using the Unified Modeling Language (UML) were constructed for each unit. The diagrams were then validated with personnel with at least 5 years of work experience at the unit. Subsequently, to elaborate a more comprehensive diagram that included the complexity of both cases observed so far, the diagrams were compared to each other considering the similarity and variability of the tasks required in each process and the resources needed to performed them. For example, Hospital 2 included an area in which the patient’s status was monitored while they remained under observation, whereas Hospital 1 did not include such a space because length of stay within the ED was less than 24 h. Hence, the pathway followed by a patient that ends up being hospitalized differs from Hospital 1 to Hospital 2.

**Table 1:** Hospitals ED included in the sample

**Institution**	**Used in**	**Characteristic**	**Medical Providers**	**Number of beds**	**Interviewee’s position**	**Years of Experience**
Hospital 1	Stage 1	High Complexity and Private Teaching Hospital.	EM Specialists, Medical interns, Residents	607	Physician	6
					Physician	8
					Nurse	10
Hospital 2	Stage 1	High Complexity and Public Teaching Hospital.	EM Specialists, Medical interns, Residents	466	Nurse	11
					Nurse	8
Hospital 3	Stage 2	High Complexity Public Hospital.	EM Specialists, Residents	340	Physician	7
					Physician	5
					Nurse	18
Hospital 4	Stage 2	Specialty High Complexity Public Hospital.	EM Specialists, Residents	176	Physician	12
					Nurse	21
					Technician	6
Hospital 5	Stage 2	High Complexity Public Hospital.	EM Specialists	211	Physician	8
Hospital 6	Stage 2	High Complexity Public Hospital.	EM Specialists, Residents	545	Physician	8
					Physician	8

The purpose of the second stage of this study was to validate the resources employed (direct and indirect costs), as well as the activities performed and services delivered (cost objects) for EDs. Using convenience sampling, four additional EDs were selected to further validate the diagram (hospitals 3 through 6 in Table 1). Table 1 shows some relevant descriptive information about the hospitals included in this study, as well as descriptive information on the healthcare professionals that were involved in the validation process.

It is important to note that Chile is an upper-middle income country located in South America. The Chilean health care system is a mixed system, which is publicly and privately financed. There is a single public insurance (FONASA) and several private health insurance companies (ISAPREs). Most of Chilean citizens (70%) are covered by FONASA. Workers can choose to be covered either by FONASA or one of the ISAPREs operating in the country. There are no barriers in FONASA to access to emergency care and highly complex pathologies. FONASA is structured in 4 groups classified by income (A to D from lower to higher income). People in groups A and B receive free health care services, group C have to pay 10% cost-sharing, and group D pay 20% of health services. In case of an emergency public beneficiaries will face the same copayments. Except for Group A, the rest have the option of using private health care facilities subscribed by FONASA. On the other hand, ISAPREs can offer different premiums to their customers to improve their health plans; most of their beneficiaries use private facilities [17]. There are copayments for people with private insurance when emergency services required exceed the plan’s ability to pay. In case of life-threatening situations, patients can receive medical attention from any ED.

In the following paragraphs we present a step by step description of the methodology executed to construct cost objects for EDs. Steps 1 through 5 correspond to stage 1 of this study, while step 6 and 7 correspond to stage 2.

### Stage 1: identifying cost objects

#### Step 1: collecting the data to define processes

Through observation and interviews with all healthcare professionals working at the two EDs included in this study at this point (hospital 1 and 2), all the processes performed within the ED were identified, considering all the tasks needed to carry them out. Each one of the tasks is considered an activity and a comprehensive list including all the activities identified (73) was elaborated. Observation occurred at different points in time (season, day of the week and time during the day) to account for seasonal effects.

#### Step 2: diagraming the processes

Using a workflow chart (using UML), all the activities identified in the previous step were diagramed considering which ones needed to be performed in sequence and which ones could be conducted in parallel. A process involves a series of activities to achieve a particular end, we identified a total of 6 processes out of 73 activities.

#### Step 3: checking the databases available to complete the process information

To make sure that all the processes needed to provide services that could be performed at the ED were considered, a list of all services accounted for each patient within the last 2 years was checked. When services provided very unfrequently were detected, the processes needed to provide them were included in the workflow charts, and therefore, the processes needed to perform them were identified.

#### Step 4: elaborating and validating processes and activities performed by the ED

All the tasks (activities) for each process were defined. The comprehensive list of activities identified, now all included in a workflow chart, were validated using the expertise of at least one physician and one nurse from the ED, preferably those that had being working at the department the longest (see Table 1). Validation of both the description and the succession of the activities occurred.

#### Step 5: defining cost objects for the ED

Once the list of identified activities was validated, groups of activities were created using three criteria. Firstly, activities were grouped according to their nature and sequence. The groups must be mutually exclusive in terms of activities, to avoid double counting when costing patients and represent 100% of the services provided by the ED. The latter translates into having combinations of groups of activities that are consumed by patients without having an overlap of activities performed, unless those activities are in actuality executed multiple times. Each one of the groups elaborated will become a cost object of the ED. Secondly, these cost objects will allow us to trace back the processes and services the ED perform. Thirdly, these cost objects can be aggregated in order to compute the costs of treatments and diseases (using any coding system). The final list of cost objects (59) corresponds to the services provided by the ED unit.

### Stage 2: validating the cost objects

#### Step 6: external validation of the activities

The final list of activities from stage 1 was validated in four EDs by healthcare professionals with expertise at the unit (see hospital 3 to 6 in Table 1).

#### Step 7: external validation of the cost objects

The final list of cost objects from stage 1 was validated in the same four EDs by the same healthcare professionals as the previous step (see hospital 3 to 6 in Table 1).

After the validation process we tested the feasibility of applying these cost objects in order to calculate costs using the ABC method at a particular ED. We chose the ABC methodology because it has being posed as a promising model for measuring costs and for making effective cost improvement decisions for the ED. We followed Kaplan & Cooper approach [18] by:

1. Developing the activity dictionary.
2. Determining how much the ED is spending on each of its activities.
3. Identifying the ED’s products, services and patients, i.e. our cost objects list.
4. Selecting activity cost drivers that link activity costs to the ED’s products, services and patients.

The list of cost objects and activities derived from this study as well as the application of the ABC method are presented in the results section.

## Results

As shown in Table 2, we identified a total of 59 cost objects for EDs. A particular ED may provide all the services listed in Table 2 or a subset of them.

**Table 2:** List of Cost Objects for EDs

**N°**	**Services**	**Description**
1	Administration of drugs by subcutaneous or intravenous injection	Administration of injectable medications of medications that can be administered to the patient without an additional approval process or paperwork because they are not controlled drugs.
2	Administration of non-injectable drugs	Administration either oral, buccal or other enteral route for non-injectable medications of medications that can be administered to the patient without an additional approval process or paperwork because they are not controlled drugs.
3	Administration of non-injectable prescribed drugs	Administration either oral, buccal or other enteral route for prescribed non-injectable medication of medications that cannot be administered to the patient without an additional approval process or paperwork because they are controlled drugs.
4	Administration of prescribed drugs by subcutaneous or intravenous injection	Administration of injectable medications of medications that cannot be administered to the patient without an additional approval process or paperwork because they are controlled drugs.
5	Alcohol screening test	Application of validated screening tools for alcohol misuse and alcohol use disorder.
6	Application of a larger orthopedic medical cast	Procedure in which a large orthopedic cast is used to treat a trauma condition.
7	Application of a shorter orthopedic medical cast	Procedure in which a short orthopedic cast is used to treat a trauma condition.
8	Arterial blood gas sample	Arterial blood gas (ABG) sampling to obtain information on a patient’s respiratory status (blood oxygen and carbon dioxide levels), as well as the patient’s acid-base balance.
9	Black braided silk suture	Procedure for approximation and/or litigation of soft tissue in which a black braided silk suture is used. This type of surgical suture is a non-absorbable multifilament composed of an organic protein.
10	Bladder instillation	Bladder instillation is a combination drug therapy to help painful bladder or cystitis type symptoms including frequency, urgency, burning pain or stinging sensations when passing urine. It works by reducing inflammation and discomfort within the bladder.
11	Blood culture sample	Blood culture collection to test for foreign invaders like bacteria, yeast, and other microorganisms in the blood.
12	Blood glucose test	Procedure to test the amount of glucose in blood.
13	Blood sampling	Blood specimen collection to obtain blood for laboratory testing.
14	Burn wound dressing	Procedure to dress a dermal burn to absorb fluid, avoid maceration and seal the wound from the outside environment to reduce pain and infection.
15	Catgut suture	Surgical suture procedure in which a catgut suture is used. This type of suture is naturally degraded by the body’s own proteolytic enzymes. It is used for surgical procedures such as general closure, ophthalmic and orthopedics.
16	Complex foreign body extraction	Complex localization, incision and removal of foreign bodies because of the location of the foreign body inside the patient’s body and the material or shape of the foreign body.
17	Complex wound dressing	Procedure to dress a complex wound that requires specialist wound care intervention. Complex wound dressing often is a result of the treatment of any number of other conditions, including cardiac, pulmonary, neuromuscular and renal diseases.
18	Diagnostic and therapeutic puncture	Medical diagnostic and therapeutic puncture for sample and treatment purposes.
19	Diagnostic puncture	Medical diagnostic puncture for sample purposes.
20	Discharge of deceased patients	It involves all the administrative processes the ED needs to follow to discharge deceased patients.
21	Electrocardiogram	Test provided at the ED to evaluate whether the patient’s heart is beating at a normal rate and strength.
**N°**	**Services**	**Description**
22	Endotracheal intubation	Endotracheal intubation is a medical procedure in which a tube is placed into the trachea, usually through the mouth, to assist breathing.
23	Enema	Procedure used to stimulate stool evacuation.
24	Feeding tube insertion	Technique in which a nasogastric tube is inserted into the patient’s nose, reaching first the back of the throat and then pushed down the esophagus until it reaches the stomach.
25	Histoacryl	Procedure in which histoacryl is applied for closing skin wounds.
26	Intramuscular drugs administration	Technique used to deliver medications deep into the muscles through an injection of medications that can be administered to the patient without an additional approval process or paperwork because they are not controlled drugs.
27	Intramuscular prescribed drugs administration	Technique used to deliver medications deep into the muscles through an injection of medications that cannot be administered to the patient without an additional approval process or paperwork because they are controlled drugs.
28	Installation of a removable cast walker boot	Procedure in which a removable cast walker boot is placed to treat trauma conditions such as severe sprains, fractures, and tendon or ligament tears in the ankle or foot.
29	IV fluid change	Procedure in which the IV fluid bag is change for another one based on the needs of the patient.
30	Life risk care management	It involves all the administrative processes the ED needs to follow to admit a patient with a life-threatening condition regardless of their ability to pay.
31	Management of imaging tests	It involves all the administrative processes the ED needs to follow to ensure that an ED patient is scheduled (if an appointment is required) for the imaging tests prescribed and transported from the ED to the Imaging Unit and back to the ED.
32	Medical attention of patients arrived by ambulance	It involves all the administrative processes the ED needs to follow to admit a patient arriving by ambulance.
33	Medical consultation	It includes the administrative process required to admit the patient into the ED, as well as the physician’s evaluation.
34	Medical interconsultation	It includes the administrative process required to ask a specialist to further evaluate the patient, in order to correctly diagnose the patient, as well as the specialist’s evaluation.
35	Medium-complex foreign body extraction	Medium-complex localization, incision and removal of foreign bodies because of the location of the foreign body inside the patient’s body or the material or shape of the foreign body.
36	Monofilament nylon suture	Procedure for approximation and/or litigation of tissue in which a monofilament nylon suture is used. This type of suture is non-absorbable by the body.
37	Nebulization	Procedure in which a nebulizer is used to treat a person with asthma or another respiratory condition to administer medication directly and quickly to the lungs.
38	Observation day	It involves all the procedures required to monitor the patient’s status while the patient remains under observation.
39	Orthopedic trauma medical attention	It includes the administrative process required to admit the patient into the ED, as well as the evaluation of the patient’s condition by an orthopedic trauma specialist.
40	Oxygen therapy	Procedure in which oxygen is provided if oxygen saturation on a patient is below the threshold defined as desirable for the patient’s condition in order to reach the targeted oxygen saturation level.
41	Phleboclysis	It corresponds to the administration of fluids intravenously drop by drop, by the drip method, through a peripheral catheter.
42	Polypropylene suture	Procedure for approximation and/or litigation of tissue in which a polypropylene suture is used. This type of suture is non-absorbable by the body.
43	Preparation of patients for hospitalization	It includes the administrative process, preparation and transference of patients to be hospitalized.
44	Preparation of patients for surgery	It includes the administrative process, preparation and transference of patients for surgery.
45	Reduction	Procedure to repair a fracture or dislocation to the correct alignment without surgery.
46	Referral	It includes all the administrative processes required to transfer a patient to another hospital, primarily because the hospital currently treating the patient has not all the resources required to successfully treat the patient’s condition.
47	Resuscitation	A life-saving procedure performed when someone has stopped breathing or the heart has stopped beating, as in cardiopulmonary resuscitation (CPR).
48	Secretion clearance	Technique aiming to remove sputum (the combination of mucus and saliva) from the patient’s lungs.
49	Simple foreign body extraction	Simple localization, incision and removal of foreign bodies because the foreign body is located in a place of the body easy to reach and the material or shape of the foreign body are not likely to cause harm while the extraction process is conducted.
50	Simple wound dressing	Procedure to dress a simple wound. This type of wound is defined as a dry dressing, apply to viable skin which does not require specialist wound care intervention.
51	Skin suture	Procedure for approximation and/or litigation of tissue using cutaneous suture.
52	Splinting	Procedure to apply a rigid or flexible device that maintains in position a displaced or movable part; also used to keep in place and protect an injured part.
53	Stomach pumping	Procedure of cleaning out the contents of the stomach through a gastric lavage.
54	Stool sample	Stool specimen collection to obtain stool (feces) for laboratory testing.
55	Teaching activities	It includes all the teaching and research activities carried out within the ED.
56	Urethral sounding	Procedure that involves inserting a sound into the urethra for dilatation of strictures or for obtaining access to the bladder.
57	Urine specimen collection	Urine specimen collection to obtain urine for laboratory testing.
58	Verify injuries	Procedures requested by a judicial order or the police to verify type and severity of injuries.
59	Vicryl suture	Procedure for approximation and/or litigation of tissue using vicryl suture. This type of suture is absorbable, synthetic, and normally braided.

Below we present the costs for all the cost objects identified in the ED from Hospital 1, using the ABC method. As shown in Table 1, Hospital 1 is a high complexity and private teaching hospital with more than six hundred beds. As a teaching hospital, patients can be treated by EM specialists, medical interns and residents. The ER at this hospital has a medical and trauma unit, as well as a critical care unit, with 10 boxes in total. The layout of this ER also includes a triage area.

**Fig. 1:**
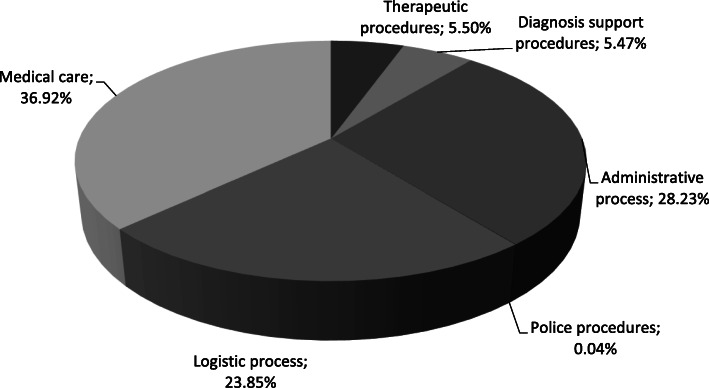
Indirect Costs of the processes from the selected ED

We calculated the cost of 53 out of the 59 cost objects that this ED provided within a calendar year. We identified and cost 6 processes and 73 activities. Figure 1 shows the indirect costs of the processes that took place in the selected ED.

As shown in Fig. 1, the medical care process has the higher proportion (36.9%) of indirect costs whereas the administrative and logistic processes consume more than half (52.1%) of the indirect resources. The indirect resources included in this ABC application can be categorized as: salaries, depreciation of equipment and infrastructure, utilities and medical supplies, office supplies, and maintenance. In order to carry out ABC, we need to recognize the tasks that make up these processes, i.e. the activities. To assign indirect resources to the activities, we define resource drivers such as time, number of procedures, number of employees/ time, usage percentage, and square meters/time.

Once activity costs were calculated, then activities were assigned to the cost objects. To allocate the cost of the activities we use the following cost drivers: time, number of procedures, and consumption index. Table 3 presents the eight most expensive activities from this ED.

**Table 3:** Eight most expensive activities (Chilean Pesos = CLP)

**Ranking**	**Activity name**	**Process name**	**Annual Cost (CLP)**
1	Medical re-evaluation	Medical care	$ 152,047,070
2	Medical evaluation	Medical care	$ 146,054,786
3	Record indications to the patient	Administrative process	$ 88,170,350
4	Patient management for imaging examination	Logistic process	$ 54,408,062
5	Management of the unit	Administrative process	$ 37,020,211
6	Clinical admission	Administrative process	$ 36,199,886
7	Withdraw supplies for procedures, meds administration and sample taking	Logistic process	$ 34,493,631
8	Take vital signs	Diagnosis support procedures	$ 29,179,405

According to Table 3, the most expensive activities are medical evaluation ($146,054,786 chilean pesos, equivalent to US$225,000) and re-evaluation ($152,047,070 chilean pesos, equivalent to US$234,000). The main reasons for the elevated costs of these activities are the physicians’ salaries and equipment and infrastructure costs.

The next step of ABC is to allocate the cost of the activities to the cost objects. The cost objects are the 53 services provided by the ED. Table 4 shows the final allocation of direct and indirect costs to these services. The services with the highest total costs are medical consultation ($487,445,524 chilean pesos, equivalent to US$750,000), phleboclysis ($97,601,148 chilean pesos, equivalent to US$150,000) and preparation of patients for hospitalization ($93,013,711 chilean pesos, equivalent to US$143,000). Eighty percent of the medical consultation costs come from three activities: medical evaluation, re-evaluation and filling health records for patients. Similarly, 80% of the phleboclysis services’ cost relates to four activities: intravenous (IV) installation, withdraws of supplies for procedures, medication administration and sample taking activities, registering medical supply consumption, and storage control. For preparation of patients for hospitalization the highest costs (78%) are associated with three activities: filling nursing and medical records for hospitalization activities, and preparing patients for transporting. However, if the unit cost is considered, the most expensive services correspond to referral ($134,507 chilean pesos, equivalent to US$207), resuscitation ($131,859 chilean pesos, equivalent to US$203), and diagnostic and therapeutic puncture ($ 43,526 chilean pesos, equivalent to US$67).

**Table 4:** Total Costs for ED Cost Objects (Chilean Pesos = CLP) including direct and indirect costs

**Ranking**	**Services**	**Indirect Costs**	**Direct Costs**	**Total Costs**	**Unit Costs**
1	Medical consultation	$ 487,445,524		$ 487,445,524	$ 11,710
2	Phleboclysis	$ 67,044,073	$ 30,557,075	$ 97,601,148	$ 7426
3	Preparation of patients for hospitalization	$ 91,101,584	$ 1,912,126	$ 93,013,711	$ 17,007
4	Management of imaging tests	$ 71,325,226		$ 71,325,226	$ 5689
5	Resuscitation	$ 37,052,388		$ 37,052,388	$ 131,859
6	Blood sampling	$ 30,124,604	$ 392,805	$ 30,517,409	$ 5795
7	Urine specimen collection	$ 18,900,132	$ 56,725	$ 18,956,857	$ 4180
8	Electrocardiogram	$ 10,555,702	$ 150,097	$ 10,705,798	$ 4603
9	Preparation of patients for surgery	$ 8,932,030	$ 438,436	$ 9,370,466	$ 7472
10	Life risk care management	$ 9,080,651		$ 9,080,651	$ 9053
11	Referral	$ 7,801,401		$ 7,801,401	$ 134,507
12	Administration of prescribed drugs by subcutaneous or intravenous injection	$ 7,655,239	$ 80,933	$ 7,736,172	$ 7130
13	Administration of noninjectable prescribed drugs	$ 7,451,112		$ 7,451,112	$ 5714
14	Monofilament nylon suture	$ 6,892,450	$ 290,026	$ 7,182,476	$ 24,265
15	Simple wound dressing	$ 6,744,059	$ 333,805	$ 7,077,865	$ 7327
16	Complex wound dressing	$ 6,889,495	$ 122,572	$ 7,012,068	$ 23,530
17	Administration of drugs by subcutaneous or intravenous injection	$ 6,201,138	$ 112,933	$ 6,314,072	$ 4170
18	Teaching activities	$ 6,225,455		$ 6,225,455	–
19	Administration of noninjectable drugs	$ 5,515,973		$ 5,515,973	$ 3032
20	Nebulization	$ 4,330,711	$ 1,005,900	$ 5,336,612	$ 4202
21	Arterial blood gas sample	$ 5,286,625		$ 5,286,625	$ 3876
22	Splinting	$ 2,187,641	$ 2,262,020	$ 4,449,661	$ 15,135
23	Blood glucose test	$ 2,945,942	$ 147,546	$ 3,093,488	$ 4701
24	Application of a shorter orthopedic medical cast	$ 1,904,884	$ 955,495	$ 2,860,379	$ 11,173
25	Installation of a removable cast walker boot	$ 289,805	$ 2,509,500	$ 2,799,305	$ 26,660
26	Orthopedic trauma medical attention	$ 2,348,362		$ 2,348,362	$ 6452
27	Black braded silk suture	$ 2,212,104	$ 80,069	$ 2,292,173	$ 24,128
28	Intramuscular prescribed drugs administration	$ 1,848,525	$ 20,737	$ 1,869,262	$ 6724
29	Medical interconsultation	$ 1,724,532		$ 1,724,532	$ 1342
30	Intramuscular drugs administration	$ 1,476,210	$ 28,942	$ 1,505,152	$ 3879
31	Application of a larger orthopedic medical cast	$ 622,032	$ 855,619	$ 1,477,650	$ 24,628
32	Stool sample	$ 1,461,081	$ 4253	$ 1,465,334	$ 4310
33	Skin suture	$ 1,280,692	$ 29,963	$ 1,310,655	$ 23,830
34	Stomach pumping	$ 983,161	$ 92,603	$ 1,075,764	$ 12,225
35	Vicryl suture	$ 745,130	$ 35,083	$ 780,212	$ 24,382
36	Histoacryl	$ 528,726	$ 10,882	$ 539,608	$ 12,549
37	Endotracheal intubation	$ 478,748	$ 23,326	$ 502,074	$ 12,246
38	Diagnostic puncture	$ 453,459	$ 21,398	$ 474,857	$ 29,679
39	Diagnostic and therapeutic puncture	$ 390,482	$ 44,779	$ 435,261	$ 43,526
40	Alcohol screening test	$ 375,863		$ 375,863	$ 19,782
41	Medical attention of patients arrived by ambulance	$ 254,133		$ 254,133	$ 12,707
42	Catgut suture	$ 209,568	$ 9610	$ 219,178	$ 24,353
43	Blood culture sample	$ 207,715	$ 8157	$ 215,873	$ 6349
44	Polypropylene suture	$ 186,282	$ 19,626	$ 205,909	$ 25,739
45	Discharge of deceased patients	$ 146,451		$ 146,451	$ 9763
46	Urethral sounding	$ 118,086	$ 12,822	$ 130,908	$ 32,727
47	Complex foreign body extraction	$ 117,215	$ 590	$ 117,806	$ 16,829
48	IV fluid change	$ 95,742	$ 5622	$ 101,364	$ 2981
49	Reduction	$ 95,091		$ 95,091	$ 5283
50	Oxygen therapy	$ 36,802	$ 23,757	$ 60,558	$ 3785
51	Verify injuries	$ 51,283		$ 51,283	$ 4274
52	Medium-complex foreign body extraction	$ 16,662		$ 16,662	$ 16,662
53	Simple foreign body extraction	$ 16,579		$ 16,579	$ 16,579

The proposed cost objects list can also be used to accumulate costs at different levels, such as patient or diagnoses. Table 5 shows an example of how the costs objects proposed can be aggregated at a diagnosis level, using the Pneumonia due to *Streptococcus pneumoniae* diagnosis (code J13 in the ICD-10 coding system).

**Table 5:** Example of costing at a diagnosis level (Chilean Pesos = CLP)

Cost Objects	**ICD-10 Code**
	**J13: Pneumonia due to*Streptococcus pneumoniae***
Medical consultation	$11,710
Arterial blood gas sample	$3876
Blood sampling	$5795
Management of imaging tests	$5689
Intramuscular prescribed drugs administration	$6724
Administration of non-injectable prescribed drugs	$5714
Nebulization	$4202
**Total Cost**	**$43,710**

As shown in Table 5, treating a patient diagnosed with Pneumonia due to *Streptococcus pneumoniae* costs $43,710 chilean pesos. This cost includes only the services provided by the ED. However, the revenue linked to those services could be imputed to the ED or to another unit at the hospital, given the classification of the services for billing purposes. In this particular case, the total revenue imputed to the ED corresponded to $19,050 chilean pesos. Hence, the ED had losses for an average of $24,660 chilean pesos per patient treated for that diagnosis in the time period included in the analysis.

The results are discussed in the next section.

## Discussion

This study proposes a methodology to identify a cost objects list for EDs that facilitates their management by improving the information available for decision-making. The analyzed case-mix allowed us to recognize 59 cost objects. This list of cost objects is better than the proposals to date because it meets with the following three design criteria: 1) Cost Objects are related to diseases, their treatments and their associated activity groups; 2) they are mutually exclusive and represent 100% of the services; 3) It allows us to trace back the processes and services provided by the ED. Hospitals may have all these cost objects or just a subset of them depending on the services they provide. The method proposed in this study can be applied to any hospital; however the final list of cost objects may differ depending on the treated cases. Moreover, the final list may end up with new cost objects based on differences in practices across countries.

Both the method to define cost objects for EDs and the cost objects list proposed are independent of the costing methodology employed. For instance, either activity-based costing or volume-based costing may be used to calculate costs. Our methodology is flexible because it recognizes activities that are aggregated according to a defined criterion to identify the final cost object list of services provided by any ED. In terms of information systems, using volume-based costing all that is required are the aggregated costs of the ED for the period under analysis, and then the selection of one driver to allocate the ED’s resources to compute the cost of the cost objects list. On the other hand, the ABC methodology used in this study allows us to aggregate the costs of activities or cost objects providing detailed information for the ED. For example, aggregating activities or cost objects at the condition level, the patient level, etc.

In comparison with previous studies, such as those using patient’s severity status as cost objects (Urgency; Emergency and Non-Emergency) [4], our proposed cost objects may both reach this level of aggregation as well as others, such as diagnoses, pre-selected diseases, case-mix and services. The same advantage can be seen when comparing to the cost objects proposal by several other authors [5, 7, 8]. Among the main benefits of our cost objects definition are: the possibility of tracing the processes generated by the services delivered by EDs, the economic sense in its grouping, the chance of using any costing methodology, the flexibility with other classification systems such as DRGs and ICDs, and the opportunity of costing for both diseases and treatments. Furthermore, cost comparison among hospitals using our final 59 cost objects list is more accurate and based on comparable units. In different EDs, each cost object will be the result of a similar combination of activities performed.

The definition of cost objects is crucial, because hospital managers can expand their analysis by focusing on continuous improvement to increase the value of care. The complexity of EDs provide a valuable setting to apply ABC given the high activity variability and hard predictability of demand. Additionally, calculating costs through ABC would help focus improvement efforts even more, for example, it would help detect which activities do not add value, optimize processes of providing services and provide more realistic cost estimates to make better managerial or strategic decisions [19].

The use of the proposed methodology makes it possible to associate cost objects with service revenue. This enables assessing the margin by each group of services. In addition, it facilitates the creation of transfer prices between different units of a hospital. In the case of the emergency unit this is especially important because many of the patients are transferred to or attended by other units. In these cases the ED does not receive the proportional compensation corresponding to the services delivered, such as, management of imaging tests, preparation of patients for surgery and medical interconsultation. Therefore, this methodology could help assess the potential economic impact of an ED within the hospital.

This study has limitations due to (1) differences in practices across institutions and countries, as well as changes in practices over time due to the development of new knowledge; and (2) sample size used for validation purposes. Even though the proposed methodology for defining cost objects is applicable to any case, the list of cost objects generated as a result of this study may not include all the services that could be provided in an ED for three reasons. First, this study analyzed a sample of six EDs. Second, technological advances could change services currently provided and make others obsolete. Lastly, epidemiological changes may demand new services.

The systematic application of this cost objects definition will enable managers to have better cost information for analysis and decision-making to avoid underfunding of EDs. Additionally, calculating these cost objects over time will allow internal comparability and benchmarking with other ED facilities. If we observed that two hospitals have a different unit cost for the same service, we may suspect that the hospital with the lower unit cost performs better than the other one. To validate the previous hypothesis, one would have to break down the difference in order to understand why those differences exist, evaluating at the same time the practices followed within each hospital. To clarify the previous argument, we can use an example. Using data from Hospital 1 and 2, we observed that the unit cost computed for medical consultation corresponds to $11,534 for Hospital 1 and to $12,268 for Hospital 2 (See Table 6). When analyzing why these difference exists, we can see that there are three activities that account for at least four-fifths of the indirect cost of the ED for both hospitals. Out of these three activities, two of them (Medical evaluation and Medical re-evaluation) consume a larger proportion of indirect costs in Hospital 1 than Hospital 2, mainly due to a larger volume of medical consultations in Hospital 1 compared to Hospital 2. For the remaining activity (Record indications to the patient), the leading cause for the discrepancy between the two hospitals is the shorter amount of time that takes to perform this activity in Hospital 1. After comparing the practices between the two hospitals, we concluded that the use of information systems to carry out the activity accounts for the minutes saved.

**Table 6:** Cost Comparison between Hospital 1 and 2 for the Medical Consultation Cost Object (Chilean Pesos = CLP)

**Activity**		**HOSPITAL 1**		**HOSPITAL 2**	
	**Cost Driver**	**Annual Cost (CLP)**	**%**	**Annual Cost (CLP**)	**%**
Request of medical advice from physician	Time	$ 8.457.972	2,0%	$ 7.249.918	1,7%
Medical evaluation	Time	$ 146.054.786	34,4%	$ 111.668.897	26,3%
Medical re-evaluation	Time	$ 152.047.070	35,8%	$ 116.224.158	27,4%
Take vital signs	Time	$ 29.179.405	6,9%	$ 25.041.997	5,9%
Call patient into the examing area	Number of patients	$ 28.583.417	6,7%	$ 24.356.080	5,7%
Record indications to the patient	Number of patients	$ 88.170.350	20,8%	$ 109.191.496	25,7%
Monitoring of clean laundry stock	Number of patients	$ 3.646.956	0,9%	$ 3.574.159	0,8%
Coordinate personnel	Time	$ 7.402.449	1,7%	$ 6.309.599	1,5%
Cleaning of the ED	Number of patients	$ 3.114.947	0,7%	$ 2.989.540	0,7%
Securty survaillance	Number of patients	$ 5.698.803	1,3%	$ 5.044.004	1,2%
Management of the unit	Number of patients	$ 15.089.370	3,6%	$ 13.171.222	3,1%
	Total Cost	$ 487.445.524		$ 424.821.069	
	Number of medical consultations	42.262		34.628	
	Unit cost	$ 11.534		$ 12.268	

Governments should encourage these types of methodologies in order to promote transparency, efficiency, and cost control through a better calculation of ED charges.

Future research could use this cost objects list to assess whether the systematic comparison of income and costs within the emergency unit, as well as between different EDs, would allow us to detect opportunities for improvement indicating which processes should be intervened.

## Conclusions

Cost objects have financial importance because they are the foundations of health insurance billing, and thus are tied to health systems financing. Different published studies use various types of cost objects for EDs. However, the lack of standardization and homogeneity in defining cost objects make comparing among units/services difficult and sometimes even impossible.

This study provides EDs with a standardized methodology to identify a list of cost objects that facilitates their management and a methodology to adapt this list to their own context. This list is better than the proposals to date and can be applied to any hospital. Hospitals may have all these cost objects or just a subset of them depending on the services they provide. Moreover, the final list may end up with new cost objects based on differences in practices across countries. This list allows to trace processes, compute costs and comparability among other EDs, provides an economic sense in its grouping, and it is flexible with any costing methodology and classification system.

## Data Availability

The datasets used and/or analysed during the current study are available from the corresponding author on reasonable request.

## Abbreviations

EDs: Emergency Departments.
DRGs: Diagnostic Related Groups
ICD: International Classification of Diseases
ABC: Activity-Based Costing
TDABC: Time-Driven Activity-Based Costing
UML: Unified Modeling Language
